# The Methanolic Extract of *Perilla frutescens* Robustly Restricts Ebola Virus Glycoprotein-Mediated Entry

**DOI:** 10.3390/v13091793

**Published:** 2021-09-08

**Authors:** Yu-Ting Kuo, Ching-Hsuan Liu, Angela Corona, Elisa Fanunza, Enzo Tramontano, Liang-Tzung Lin

**Affiliations:** 1 Department of Medical Imaging, Chi Mei Medical Center, Tainan 710, Taiwan; y.kuo@mail.chimei.org.tw; 2 Department of Microbiology and Immunology, School of Medicine, College of Medicine, Taipei Medical University, Taipei 110, Taiwan; d119107007@tmu.edu.tw; 3 Department of Life and Environmental Sciences, University of Cagliari, Monserrato, 09124 Cagliari, Italy; angela.corona@unica.it (A.C.); elisafanunza@live.it (E.F.); tramon@unica.it (E.T.); 4 Graduate Institute of Medical Sciences, College of Medicine, Taipei Medical University, Taipei 110, Taiwan

**Keywords:** Ebola virus, glycoprotein, natural product, *Perilla frutescens*, viral entry inhibition

## Abstract

Ebola virus (EBOV), one of the most infectious human viruses and a leading cause of viral hemorrhagic fever, imposes a potential public health threat with several recent outbreaks. Despite the difficulties associated with working with this pathogen in biosafety level-4 containment, a protective vaccine and antiviral therapeutic were recently approved. However, the high mortality rate of EBOV infection underscores the necessity to continuously identify novel antiviral strategies to help expand the scope of prophylaxis/therapeutic management against future outbreaks. This includes identifying antiviral agents that target EBOV entry, which could improve the management of EBOV infection. Herein, using EBOV glycoprotein (GP)-pseudotyped particles, we screened a panel of natural medicinal extracts, and identified the methanolic extract of *Perilla frutescens* (PFME) as a robust inhibitor of EBOV entry. We show that PFME dose-dependently impeded EBOV GP-mediated infection at non-cytotoxic concentrations, and exerted the most significant antiviral activity when both the extract and the pseudoparticles are concurrently present on the host cells. Specifically, we demonstrate that PFME could block viral attachment and neutralize the cell-free viral particles. Our results, therefore, identified PFME as a potent inhibitor of EBOV entry, which merits further evaluation for development as a therapeutic strategy against EBOV infection.

## 1. Introduction

Ebola virus (EBOV), the etiologic agent of Ebola virus disease (EVD), is an enveloped negative-sense single-stranded RNA virus, belonging to the *Filoviridae* family. This family of viruses also includes the Marburg virus and is characterized by a thread-like, filamentous morphology [1]. There are six species of ebolaviruses, namely Zaire (EBOV), Sudan, Bundibugyo, Taï Forest, and Reston, which are known to infect humans, and Bombali, for which there is no confirmed human case to date [2]. EBOV infection is a leading cause of viral hemorrhagic fever in human and nonhuman primates (NHP), with average case mortality of 50–90% [3]. Since its emergence in 1976, EBOV has caused sporadic outbreaks that were mostly restricted to parts of Africa, until the unprecedented 2013–2016 regional epidemic, which started in Guéckédou, Guinea and spread to various countries, thereby imposing a public health emergency of international concern [4]. Several recent localized outbreaks have also occurred between 2018 and 2021, including the 2021 epidemics in the Democratic Republic of Congo [5] and Guinea [6]. These recent outbreaks prompted an urgency for further pushing the ongoing vaccine and antiviral development, and it was not until December 2019 that immunization became possible with the U.S. FDA-approved rVSV-ZEBOV vaccine (Ervebo) [7]. More recently, the combination monoclonal antibody-based treatment Inmazeb (previously REGN-EB3), was also approved by the U.S. FDA in October 2020, making it the first licensed antiviral treatment against EBOV [8]. Such outcomes are encouraging, due to the difficulty of working with this risk group 4 pathogen, and highlight the importance of undeterred and continuous research development of clinical strategies for the prevention and treatment of EBOV infection. This includes identifying antiviral agents that target EBOV entry, which could improve the therapeutic arsenal against the deadly filovirus and could play an important role in cases where full scale immunization coverage is not possible.

The genome of EBOV is 19 kb in length and encodes seven viral proteins, including the nucleoprotein (NP), polymerase cofactor (VP35), matrix proteins (VP40 and VP24), the transcription activator (VP30), RNA polymerase (L), and the glycoprotein GP. GP is the sole viral protein expressed on the surface of the viral particle and constitutes the main viral protein responsible for EBOV entry, making it an attractive target for therapeutic interventions, such as the design of vaccines and entry inhibitors against the virus. GP is post-translationally cleaved by the furin protease, yielding two disulfide-linked subunits, GP1 and GP2, which respectively orchestrate viral attachment to host cells and fusion of the viral and host cell membranes [9]. EBOV enters host cells through macropinocytosis and possibly other endocytic pathways [10,11], and further processing of GP by the endosomal cathepsins, such as cathepsins L and B [12], which expose the GP receptor binding site to facilitate its binding to the Niemann-Pick C1 (NPC-1) host cell receptor in the endosome [13,14], are believed to be required for its entry [15].

Viral entry, the first step of the viral life cycle, constitutes an attractive target for prophylactic/therapeutic interventions since blocking viral entry impedes the subsequent steps of the viral life cycle. Entry inhibitors have emerged as an essential class of antiviral agents that have been used against viral infections, such as Maraviroc against human immunodeficiency virus (HIV) [16], Bulevirtide against hepatitis D virus [17], and the more recently approved Inmazeb against EBOV [8]. As an effort to expand the scope of antiviral candidates targeting EBOV entry, herein we employ EBOV pseudoparticles (EBOVpp), which are biosafety level (BSL)-2-safe viral particles expressing recombinant EBOV GP and previously shown to allow identification of entry inhibitors against the virus [18,19], to screen a panel of natural medicines that serve as an excellent source of antiviral discovery [20]. We describe in the following, our identification of the methanolic extract of *Perilla frutescens* (PFME) as a plant-derived antiviral candidate with potent anti-EBOV entry activity.

## 2. Materials and Methods

### 2.1. Reagents

Dulbecco’s modified Eagle’s media (DMEM; #11995040), fetal bovine serum (FBS; #26140079), and gentamicin (#15750060) were purchased from GIBCO/ThermoFisher Scientific (Waltham, MA, USA). Dulbecco’s phosphate-buffered saline (DPBS; #SH30028.03) was obtained from Hyclone (Logan, UT, USA). Dimethyl sulfoxide (DMSO; #SI-D5879), polyethylene glycol 8000 (PEG-8000; #SI-P1458), and amphotericin B (#A2942) were purchased from Sigma-Aldrich (Saint Louis, MO, USA).

### 2.2. Cells and Plasmids

Human hepatoma cells Huh-7 were cultured in DMEM supplemented with 10% FBS, 1% gentamycin, and 1% amphotericin B, and incubated at 37 °C in 5% CO_2_ incubator as described previously [21]. Human embryonic kidney HEK 293FT cells (Invitrogen/Thermo Fisher Scientific) were cultured as above but supplemented with 0.5 mg/mL G418 sulfate (InvivoGen; San Diego, CA, USA). For all viral infections, a basal medium of DMEM containing 2% FBS and antibiotics was used. For EBOVpp production, the HIV-1-based lentiviral vector containing a firefly luciferase reporter tag has previously been described [22]. The EBOV GP expressor sequence is generated based on the Mayinga strain of Zaire EBOV (accession number U23187.1) through gene synthesis and cloned into pcDNA3.1 (Invitrogen/ThermoFisher Scientific) using standard cloning techniques.

### 2.3. Production of EBOVpp

The production of EBOVpp was carried out using a previously described method for generating lentivirus-based pseudoparticles with some modifications [23]. 5 × 10^6^ HEK 293FT cells were seeded in 10-cm dishes overnight, prior to co-transfection next day with 10 μg lentiviral vector and 10 μg EBOV GP plasmids using OMNIfect transfection reagent (Transomic Technologies; Huntsville, AL, USA). The cells were then incubated at 37 °C in 5% CO_2_ incubator overnight before removing the transfection complexes with DPBS washes and overlaying the cells with complete DMEM for further incubation. The culture supernatants were collected at 72 h and 96 h post-infection, clarified by centrifugation, and then concentrated using PEG-8000 as previously described [23], before the virus pellet was resuspended in DPBS and stored at −80 °C.

### 2.4. Luciferase Luminescence-Based TCID50 Virus Titre Quantification

Huh-7 cells (1 × 10^5^ cells/well of 48-well plates) were infected with EBOVpp at 10-fold serial dilutions in triplicate for 2 h at 37 °C. After which, the cells were washed by DPBS and replaced with basal media for 72 h incubation at 37 °C. The cells were then harvested and assessed using the Firefly Luciferase Assay kit and a luminometer (Promega; Madison, WI, USA) according to the manufacturer’s protocol. The luciferase reporter signal corresponding to viral infectivity from successful EBOVpp entry was then used to determine the 50% tissue culture infectious dose (TCID_50_) based on the Reed-Muench method [24]. Wells were scored as positive when the reporter signal was at least 1 log_10_ higher than the mock controls.

### 2.5. Western Blot

To detect EBOVpp production, purified EBOVpp were processed using RIPA buffer (Sigma) supplemented with cOmplete Mini Protease Inhibitor Cocktail (ROCHE; Basel, Switzerland) and the protein concentrations were determined using the Bio-Rad Protein Assay kit (Bio-Rad Laboratories; Hercules, CA, USA). Following which, samples were resolved by sodium dodecyl sulfate-polyacrylamide gel (SDS-PAGE) electrophoresis and transferred onto a nitrocellulose membrane by standard Western blotting techniques. The blots were then probed using the primary anti-EBOV GP (subtype Zaire, strain Mayinga 1976) rabbit polyclonal antibody (Sino Biological; Beijing, China) at 1:1000 dilution, and HIV-p17 monoclonal antibody (Santa Cruz Biotechnology; Dallas, TX, USA) at 1:200 dilution, followed by the respective horseradish peroxidase (HRP)-conjugated secondary antibodies (Abcam; Cambridge, United Kingdom) at 1:3000 dilution and detection using ECL chemiluminescent substrate (Bio-Rad Laboratories). Images were taken with a ChemiDoc-It^TS2^ imager (UVP; Upland, CA, USA).

### 2.6. Transmission Electron Microscopy

HEK 293FT cells (5 × 10^6^ cells seeded in 10-cm dishes) were transfected for EBOVpp production as described above and transferred to Lab-Tek II chamber slides (Nunc/ ThermoFisher Scientific) for 72 h incubation at 37 °C. The cells were subsequently fixed with 2% paraformaldehyde and 2.5% glutaraldehyde in 0.2 M cacodylate, then rinsed with 0.2 M cacodylate plus 7% sucrose for 45 min, before Osmium tetroxide (OsO_4_) fixation for 2 h. The samples were next rinsed again with 0.2 M cacodylate plus 7% sucrose for 45 min, and then dehydrated with different percentages (70–100%) of alcohol for 2 h. Afterwards, the samples were treated with propylenoxid for 30 min, then fixed with epichlorohydrin and propylenoxid for 24 h, followed by incubation at 62 °C for 72 h. All chemical reagents used in the sample preparation are of analytical grade. The samples were finally analyzed with a transmission electron microscope (TEM HT7700; HITACHI, Tokyo, Japan).

### 2.7. Drug Preparation

The candidates used for antiviral screening are aqueous and methanol extracts from referenced medicinal plant materials [25] and were kindly provided by Dr. Ming-Hong Yen (Kaohsiung Medical University, Kaohsiung, Taiwan). The plant materials were freshly obtained from a local pharmacy store (Kaohsiung, Taiwan), authenticated by anatomical methods and HPLC analysis, and extracted using standard hot-water [26] and methanol [27] extraction methods. The water-soluble extracts were reconstituted using ddH_2_O, and the methanol extracts were reconstituted in DMSO. All extracts were diluted with culture media to their final working concentrations consisting ≤0.01% DMSO.

### 2.8. Cytotoxicity Assay

Huh-7 cells (1 × 10^4^ cells/well seeded in 96-well plates) were treated with the different drug candidates at various concentrations for 72 h incubation at 37 °C, before cytotoxicity analysis was performed using the cell counting kit-8 (CCK-8) kit (Sigma). CCK-8 reagent was added to the drug-treated cells for 2 h incubation at 37 °C, before optical densities at 450 nm were measured using the SpectraMax Plus 384 Microplate Reader (Molecular Devices, LLC.; San Jose, CA, USA) to determine the cell viability percentage and the 50% cellular cytotoxicity (CC_50_) as previously described [28].

### 2.9. EBOVpp Antiviral Screening

Huh-7 cells (1 × 10^5^ cells/well seeded in 48-well plates) were first pre-incubated with the different drug candidates at their respective maximal non-cytotoxic dose for 24 h at 37 °C. Following which, the cells were washed with DPBS, and then inoculated with EBOVpp (multiplicity of infection (MOI) 0.01) concurrently with the addition of each extract (at their respective initial concentration used) for 2 h at 37 °C. The virus–drug inoculum was next removed and cells were washed using DPBS and then incubated in basal media for 72 h at 37 °C before luciferase assay analysis as described above.

### 2.10. Dose-Response Assay

Huh-7 cells (1 × 10^5^ cells/well seeded in 48-well plates) were pre-incubated with the drug candidate at the indicated doses for 24 h, and then infected with EBOVpp (MOI 0.01) in the presence of the drug candidate again at the same concentration range. After 2 h infection at 37 °C, the cells were washed with DPBS and further incubated in basal media for 72 h at 37 °C, before luciferase assay was performed as described earlier.

### 2.11. Time-of-Drug-Addition Assay

The time-of-drug-addition assay was performed as described previously [29] with modifications. The test drug agent was added to Huh-7 cells (1 × 10^5^ cells/well seeded in 48-well plates) either for 24 h before (pretreatment) or concurrently with (co-addition) a 2 h-infection with EBOVpp (MOI 0.01) at 37 °C. Wash steps were included to ensure that the drug or virus was only present during the specific incubation period. Following 72 h incubation post infection, the cells were washed with DPBS before performing the luciferase assay analysis as described above.

### 2.12. Synchronized Infection Assay

The synchronized infection assay was performed to identify the test drug’s effect on different stages of viral entry using previously reported method [30] with modifications. (i) Viral inactivation assay: EBOVpp was first pre-incubated with the test drug agent in test tube for 2 h at 37 °C. The virus–drug mixture was then diluted 6-fold to ineffective concentration of the drug, before being used to infect Huh-7 cells (1 × 10^5^ cells/well seeded in 48-well plates) at a final MOI of 0.01 for 2 h at 37 °C. The infection inoculum was next removed and the cells were washed using DPBS, then further incubated in basal media for 72 h. (ii) Viral attachment assay: Huh-7 cells (1 × 10^5^ cells/well seeded in 48-well plates) were first pre-chilled at 4 °C for 1 h, then incubated with EBOVpp (MOI 0.01) in the presence of the test drug agent for 2 h at 4 °C to permit only virus binding but not penetration into the host cell, before removing the virus–drug inoculum with ice-cold DPBS wash. The virus-bound cells were subsequently covered in basal media and incubated at 37 °C, which allows viral penetration, for 72 h. (iii) Viral entry/fusion assay: Huh-7 cells (1 × 10^5^ cells/well seeded in 48-well plates) were first pre-chilled at 4 °C for 1 h, and then infected with EBOVpp (MOI 0.01) for 2 h at 4 °C to permit only viral attachment but not penetration. The virus inoculum was next removed with ice-cold DPBS wash, and the virus-bound cells were treated with the test drug agent for 2 h incubation at 37 °C which allows viral fusion/penetration. The drug-containing media was later removed by DPBS washes, and the cells were further incubated in basal media at 37 °C for 72 h. For (i) and (iii), the anti-EBOV GP KZ52 antibody (25 μg/mL; Integrated BioTherapeutics, Rockville, MD, USA), which can neutralize EBOV GP and prevent EBOV infection [31] by specifically targeting cathepsin cleavage in the viral fusion step [32], was included as a positive control. For all assays, the cells were harvested for analysis by luciferase assay at 72 h post infection, as described above.

### 2.13. Statistical Analysis

GraphPad Prism 8 software (San Diego, CA, USA) was used for quantitative analysis. Data were represented as means ± standard deviation (SD), and statistical analysis was performed using one-way ANOVA with Dunnett post hoc test. A *p*-value of <0.05 was considered statistically significant.

## 3. Results

### 3.1. Production and Characterization of EBOVpp

**Figure 1:**
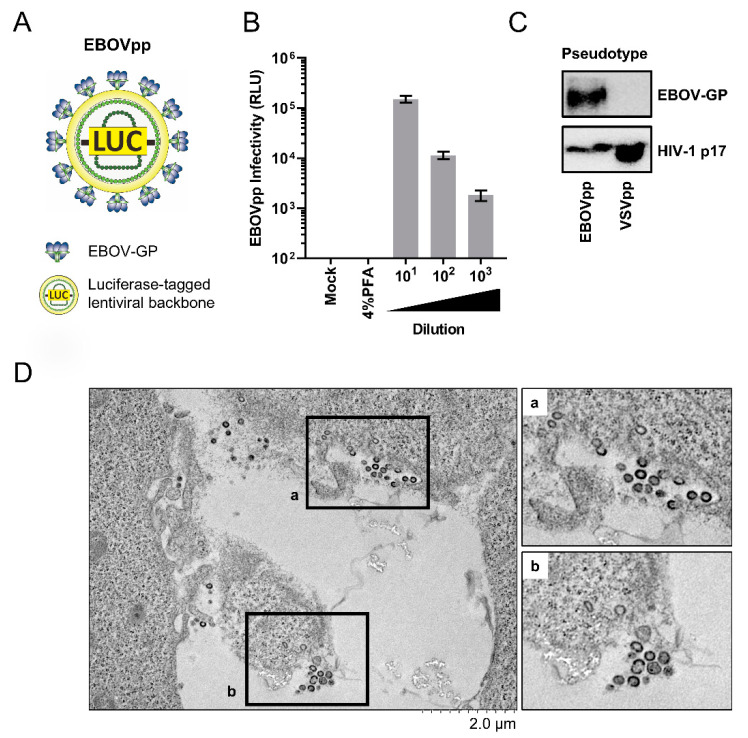
Production and Characterization of EBOVpp. (**A**) Schematic of EBOVpp with surface-expressing EBOV-GP and an HIV-based luciferase reporter-tagged lentiviral backbone. (**B**) Infectivity analysis and titration of EBOVpp (10-fold serial dilution) on Huh-7 cells. Luciferase reporter activity was determined at 72 h post-infection to determine viral infectivity. Paraformaldehyde (PFA; 4%) treatment, which fixes the cells and renders them impermeable to virus internalization, was included as a negative control. Data are expressed as mean relative light units (RLU) ± SD from 3 independent experiments. (**C**) Analysis of EBOV GP expression in supernatant-harvested pseudoparticle preparation by western blotting. Images shown are representative blots from three independent experiments. VSV-G pseudoparticles (VSVpp) are included for comparison and anti-HIV-p17 matrix protein served as control. (**D**) Transmission electron microscope (TEM) imaging of EBOVpp release from transfected HEK 293FT cells. Representative micrographs from three independent experiments are shown.

To identify novel entry inhibitors against EBOV, we utilize EBOVpp which were produced by transfecting HEK 293FT cells with plasmids encoding the EBOV GP (Mayinga strain) and a luciferase reporter-tagged HIV-1-based lentiviral vector, yielding chimeric viral particles displaying EBOV GP on a lentiviral core (Figure 1A). The generated EBOVpp were then used to infect and titrate on the liver-derived Huh-7 cells. Wash steps following infection and paraformaldehyde treatment control (which fixes the cells and prevents infection) were included to ensure that the reporter signals were not due to carry-over luciferase from the virion inoculum. As shown in Figure 1B, EBOVpp successfully infected Huh-7 cells, yielding luciferase signals indicative of successful viral entry mediated by EBOV-GP and that could be serially titrated and demonstrated a linear correlation with viral titer. This observation is consistent with published literature reporting usage of Huh-7 as a permissible host cell line to EBOV infection [33,34,35,36]. Further western blot analysis confirmed the production of authentic EBOVpp, whereby in contrast to detecting the HIV-1 matrix protein p17 across the samples of pseudoparticles bearing vesicular stomatitis virus (VSV) glycoprotein G or EBOV GP, EBOV GP was only detected in the EBOVpp (Figure 1C). Furthermore, transmission electron microscope (TEM) imaging showed spherical particles typical of HIV virions due to the HIV-derived core in the EBOVpp as opposed to the filamentous morphology of wilt-type EBOV particles (Figure 1D). Together, these results confirmed successful production of the EBOVpp for our antiviral screening experiments.

### 3.2. Screening of Plant-Derived Natural Medicines Identified the Methanolic Extract of Perilla frutescens (PFME) as an Antiviral Candidate against EBOV Entry

**Figure 2:**
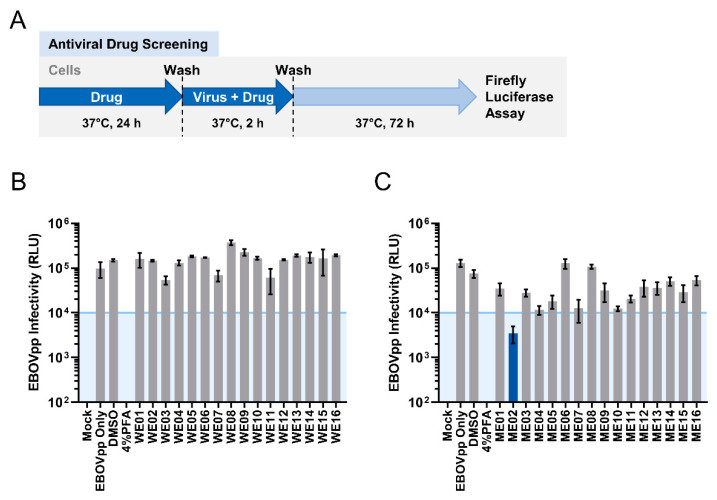
Antiviral Screening of Natural Medicine Candidates against EBOVpp Infection. (**A**) Schematic of EBOVpp antiviral screening procedure. Antiviral efficacy of the water extracts (WE) (**B**) and methanolic extracts (ME) (**C**) of each natural medicine candidate against EBOVpp (MOI = 0.01) infection on Huh-7 cells. DMSO (0.01%) and 4% PFA treatments are included as controls. Data are expressed as mean RLU ± SD from three independent repeats. The 10^4^ threshold is shown by blue line with shade.

The biodiversity of plants and their phytochemical contents represent an excellent source of novel antiviral drug discovery. We next therefore picked a panel of heat-clearing and detoxicating medicinal herbs [25], and screened their water and methanol extracts as candidates for anti-EBOV entry inhibition activity. The test drug agents were first evaluated for cytotoxicity on Huh-7 cells to determine their CC_50_ as well as their maximum non-cytotoxic (≥95% cell survival)/screening concentrations (Table 1). To examine the anti-EBOV entry activity of the candidates, we pre-incubated Huh-7 cells with each of the test product at its screening concentration for 24 h, before infecting the cells with MOI 0.01 of EBOVpp in the presence of the test drug agents for 2 h as illustrated in Figure 2A. We then evaluated EBOVpp infection based on the levels of luciferase reporter signal and defined 10^4^ relative light units (RLU) as the minimum threshold level (approximately one-log reduction compared to their non-treated controls) for the selection of potential EBOV antiviral candidates (Figure 2B,C). While none of the water extracts achieved prominent anti-EBOV entry activity (Figure 2B), a few methanolic extract candidates showed anti-EBOV inhibition (Figure 2C). Specifically, the methanolic extract (ME02) of *Perilla frutescens* (PFME) demonstrated the strongest effect with the most substantial decrease (~1.5 log) in the luciferase reporter activity compared to the DMSO control (Figure 2C). Therefore, PFME was chosen as our priority anti-EBOV candidate for all subsequent experiments.

### 3.3. PFME Blocks the Early Entry Steps of EBOVpp Infection

**Figure 3:**
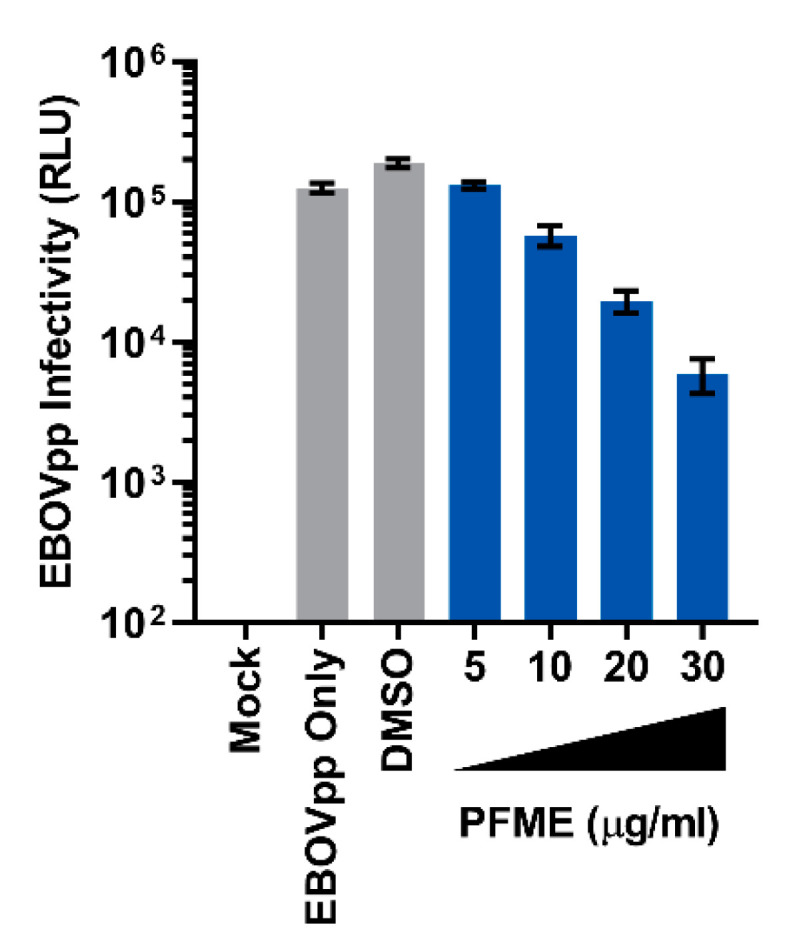
Dose-Response Analysis of PFME against EBOVpp Infection. Dose-response of PFME using the indicated concentrations against EBOVpp (MOI 0.01) infection on Huh-7 cells. Luciferase reporter assay was performed at 72 h post-infection to evaluate viral infectivity. DMSO (0.01%) served as a negative control. Data are expressed as mean RLU ± SD from three independent repeats.

**Figure 4:**
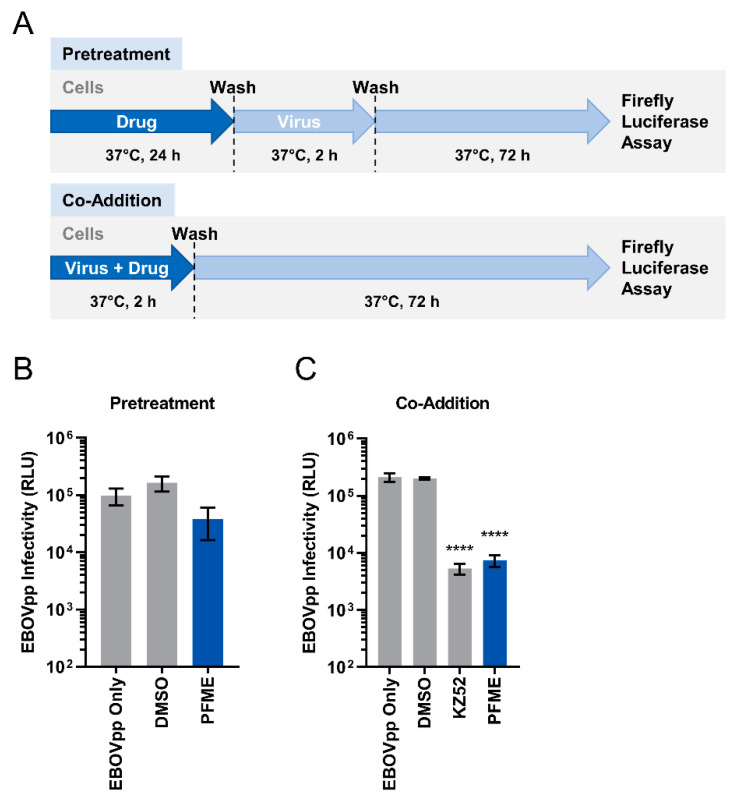
Time-of-Drug-Addition Analysis of PFME against EBOVpp Infection. (**A**) Schematic of time-of-drug-addition analysis of PFME (30 μg/mL) treatment against EBOVpp (MOI 0.01) infection on Huh-7 cells using (**B**) pretreatment and (**C**) co-addition models. For the co-addition assay, EBOV GP-neutralizing antibody KZ52 [31] was included as a positive control. For both experiments, DMSO (0.01%) treatment served as negative control. Luciferase reporter assay was performed at 72 h post-infection to assess EBOVpp infection. Data are expressed as mean RLU ± SD from three independent experiments. Asterisks (*) denote statistical significance: **** *p* < 0.0001.

The anti-EBOV activity of PFME was first studied via a dose-response assay. We pretreated Huh-7 cells with increasing concentrations (5–30 μg/mL) of the extract for 24 h before infecting the cells with MOI 0.01 of EBOVpp again in the presence of PFME at the different concentrations. We found that PFME dose-dependently inhibited EBOVpp infection (Figure 3). Next, we investigated how PFME impedes EBOVpp infection by performing a time-of-drug-addition assay, wherein cells were either pretreated with PFME for 24 h before washing and infection (pretreatment) or infected in the presence of PFME (co-addition), as depicted schematically in Figure 4A. Our results showed that 30 μg/mL of PFME in co-addition treatment (Figure 4C) significantly reduced EBOVpp infection by over 1 log, in contrast to pretreatment wherein PFME exerted only minor but non-statistically significant effect (Figure 4B). These results suggest that PFME likely targets the early phases of EBOVpp entry during its infection.

### 3.4. PFME Blocks EBOVpp Attachment and Neutralizes the Viral Particles

**Figure 5:**
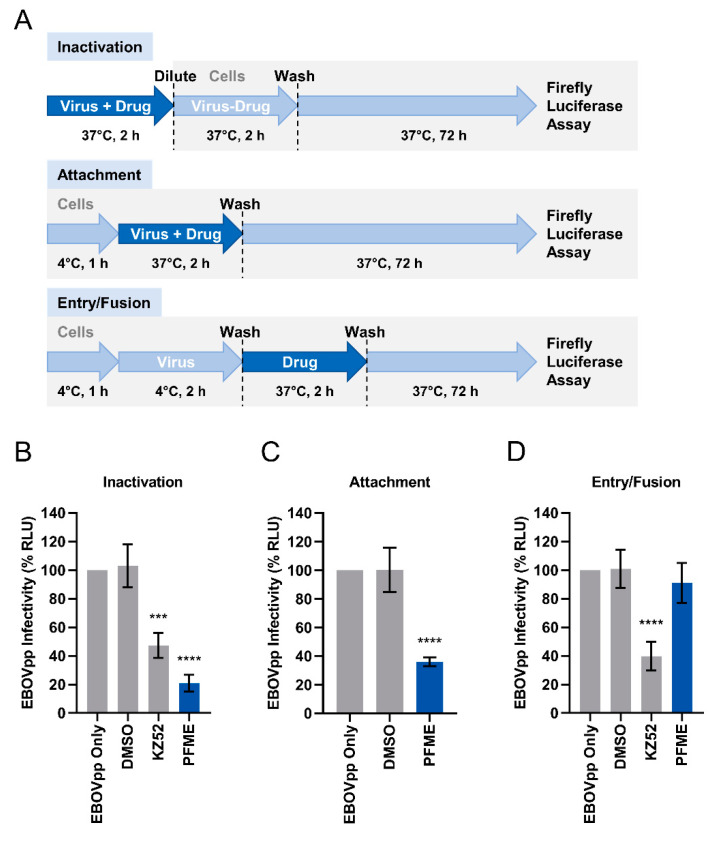
PFME Effectively Inactivates EBOVpp and Blocks Viral Attachment to Host Cells. (**A**) Schematics of the synchronized infection assay on early viral entry. (**B**) Effect of PFME (30 μg/mL) treatment on cell-free EBOVpp (final MOI = 0.01). (**C**) Effect of PFME (30 μg/mL) treatment on EBOVpp (MOI 0.01) viral attachment. (**D**) Effect of PFME (30 μg/mL) treatment on EBOVpp (MOI 0.01) viral entry/fusion. Anti-GP antibody KZ52 (25 μg/mL), which specifically prevents the formation of fusion-activated EBOV GP [32], was included as a positive control for inactivation and entry/fusion assays. Luciferase reporter assay was performed at 72 h to evaluate EBOVpp infectivity (%). DMSO (0.01%) treatment served as negative control. Data are expressed as mean ± SD from three independent experiments. Asterisks (*) denote statistical significance: *** *p* < 0.001; **** *p* < 0.0001.

Since the most robust antiviral impact of PFME against EBOVpp entry appear to occur when both the EBOV GP-pseudotyped viral particles and the test drug are concurrently present on the host cell (Figure 4C co-addition scenario), we hypothesized that PFME possibly act by influencing the early viral entry steps mediated by the EBOV GP. To decipher which specific step(s) of the early viral entry event is targeted by PFME, we performed a synchronized infection assay in which we examined whether PFME inactivates cell-free EBOVpp from being able to bind target cells to initiate infection, or PFME inhibits viral attachment to host cell receptor, or PFME inhibits post-attachment viral fusion with host cell (Figure 5A). The KZ52 neutralizing antibody, which binds to EBOV GP [31] and prevents cathepsin cleavage required for the viral fusion step [32], was included as a positive control for inactivation and entry/fusion assays. Our findings show that 30 μg/mL PFME potently inactivated free viral particles, with better neutralizing activity than the KZ52 neutralizing antibody positive control [31], and diminished EBOVpp infection (Figure 5B). In addition, 30 μg/mL PFME also blocked EBOV attachment to the host cells and suppressed the infection (Figure 5C). In contrast, PFME did not exert a significant effect on viral entry/fusion (Figure 5D), whereas the KZ52 antibody treatment effectively blocked this step. These results indicate that PFME mediates its anti-EBOVpp activity likely by neutralizing the free viral particles and blocking viral attachment to the host cells. Overall, we identified and propose PFME as an efficient novel antiviral inhibitor candidate against EBOV-GP-mediated viral entry.

## 4. Discussion

The past several decades has witnessed the emergence and re-emergence of viral pathogens including EBOV, underscoring the need for continuous development of vaccination and therapeutic strategies to curtail these viruses and prevent their spread. Due to its highly infectious nature and the lack of antiviral therapeutics that were only recently approved, the deadly EBOV and its associated EVD represent an important public health threat to be addressed urgently in preparation for potentially devastating future outbreaks. Identifying antiviral agents that restrict EBOV entry into host cells and prevent establishment or limit propagation of its infection are advantageous as they could serve not only as therapeutic drugs, but also for prophylaxis purposes (pre- or post-exposure) which is key in reducing viral transmission and providing protection for high-risk healthcare workers [37]. Indeed, entry inhibitors have emerged as an important class of antiviral agents for the prevention/treatment of various viral infections and have been explored widely, including against HIV-1 [38,39,40,41], herpes simplex virus [28,42], avian influenza [43,44], dengue virus [45,46], hepatitis C virus [23,26,47,48,49], measles virus [50,51], and enteroviruses [52,53]. These developments prove the utility and significance of entry inhibitors as a vital class of antiviral agents. Although entry inhibitors in principle do not stop post-entry viral replication, they are useful to restrict the *de novo* infection spread by progeny viruses, and their combination with other antiviral agents can enhance antiviral efficacy and help prevent the emergence of drug resistance, as evidenced by the inclusion of entry inhibitors in the highly active antiviral therapy (HAART) against HIV-1 [54], as well as experimental combination therapy with direct-acting antivirals (DAAs) for HCV [55]. Similarly, antiviral therapeutics blocking EBOV entry have been examined in the past decades, including neutralizing monoclonal antibodies (ZMapp, mAb 114, REGN-EB3) [56,57,58], synthetic chemicals (diaryl-quinoline compounds, sulfonamide MBX2254, triazole thioether MBX2270, cationic amphiphiles, G protein-coupled receptor antagonists) [19,59,60,61], and natural products such as quercetin 3-β-d-glucoside [62] and sclareol and sclareolide from the plant *Salvia sclarea* [63]. Our finding that PFME efficiently suppresses EBOV GP-mediated entry adds to the growing list of entry inhibitory agents against EBOV that deserve further evaluation for development as EBOV entry inhibitors.

Our study demonstrated that 30 μg/mL PFME co-addition most effectively inhibited EBOVpp infection (Figure 4C), suggesting that the extract likely targets the early viral entry events of the virus. To elucidate the specific entry-related step that PFME targets to block EBOVpp entry, we performed a synchronized infection assay on the early viral entry steps, and found that 30 μg/mL PFME strongly inactivates cell-free EBOVpp (Figure 5B). In addition, compared to the EBOV GP-neutralizing antibody KZ52, PFME had higher inhibition rate, indicating that the extract possesses strong neutralizing capacity against EBOVpp infectivity. Besides this, PFME also robustly blocked viral attachment to the host cells (Figure 5C), but had no significant effect on post-binding viral entry/fusion (Figure 5D). Therefore, we speculate that the antiviral activity of PFME appears to precede the endosomal cathepsin cleavage steps, and may involve modifying EBOV GP to (1) prevent receptor interaction (hence rendering the EBOVpp particle inactive) and/or (2) to preclude the subsequent GP conformational change from the required protease cleavage for binding to NPC1 in the endosome that leads to release of the viral genome [15]. However, we could not completely rule out that PFME may potentially also modulate host cell factors required for viral entry to block the EBOVpp infection. For instance, the presence of PFME on the cells could downregulate expression of the host cell factors that facilitate EBOV attachment and entry (β1 integrins, folate receptor-α, C-type lectins, T-cell immunoglobulin and mucin domain 1, and Tyrosine kinase receptor Axl) [64,65,66,67,68,69,70,71,72], modulate host macropinocytosis that promotes EBOV particle engulfment [10,73], or boost the innate antiviral immune response which is known to block viral infection [74]. A preliminary analysis of the cell innate immune response to PFME treatment showed that the extract can moderately stimulate interferon (IFN)-β promoter activation (~0.6 fold over control), which could be suppressed by the overexpression of the IFN-inhibiting EBOV VP35 [75] (Figure S1). Further in-depth analysis on host cell attachment factors, macropinocytosis pathway, and cell innate immune response may help to fully clarify the spectrum of antiviral activity of PFME.

Although PFME showed promising inhibition against EBOV GP-mediated viral entry, the specific component of the extract that mediates its antiviral activity remains to be elucidated. In an attempt to identify the active compounds responsible for PFME’s anti-EBOV activity, we tested the effect of rosmarinic acid, which is previously reported as the dominant antioxidant phenolic compound in PFME, against EBOVpp entry [76,77]. Our preliminary results did not reveal significant antiviral activities from rosmarinic acid (Figure S2), indicating that perhaps other less abundant active components or a combination of these compounds may be responsible for the extract’s antiviral activity. Further research would be needed to pinpoint the exact component(s) of PFME responsible for its anti-EBOV entry activity. In addition, while we have used the EBOVpp as an in vitro system to identify PFME as a potential antiviral candidate blocking EBOV entry, further studies should be explored in vivo for testing drug bioavailability and efficacy.

In summary, using the EBOVpp system, we identified the natural extract PFME as an efficient inhibitory agent of EBOV entry that acts by neutralizing the cell-free viral particles and blocking viral attachment to the host cell. We suggest that PFME is of value for further drug development as an anti-EBOV therapeutic agent.

## Figures and Tables

**Table 1:** List of Natural Medicine Candidates and Their CC_50_ and Screening Concentration Used.

ID	Species	Part(s)	CC_50_ (μg/mL)		SC (μg/mL)	
			ME	WE	ME	WE
01	*Artemisia annua*	Herba	237	>800	46	<1
02	*Perilla frutescens*	Folium	169	>800	30	100
03	*Zingiber officinale*	Rhizome	40	>800	4	800
04	*Schizonepeta tenuifolia*	Herba	693	>800	135	50
05	*Mentha canadensis*	Herba	327	>800	70	100
06	*Chrysanthemum morifolium*	Flos	1011	>800	27	500
07	*Morus alba*	Folium	460	>800	83	500
08	*Saposhnikovia divaricate*	Radix	987	>800	251	800
09	*Cimicifuga foetida*	Rhizome	47	>800	12	100
10	*Isatis indigotica Fort.*	Folium	663	>800	131	100
11	*Polygonum cuspidatum*	Radix	120	621	26	100
12	*Dryopteris crassirhizoma*	Rhizome	1575	>800	420	500
13	*Anemarrhena asphodeloides*	Rhizome	730	>800	294	500
14	*Sophora tonkinensis*	Radix	287	>800	17	10
15	*Aster tataricus*	Radix et rhizome	818	>800	74	10
16	*Artemisia argyi*	Folium	212	555	29	50

CC_50_ and screening concentration (SC) of the candidate extracts as determined by CCK-8 cell viability assay. Data represent mean from three independent experiments. ME = methanol extract; WE = water extract.

## Data Availability

The data presented in this study are available in the article and Supplementary Materials.

## Supplementary Materials

The following are available online at https://www.mdpi.com/article/10.3390/v13091793/s1, Figure S1: PFME Induces IFN-β Promoter Activation That Can Be Suppressed by the Overexpression of the IFN-β Antagonist EBOV VP35, Figure S2: Rosmarinic Acid Does Not Impede EBOVpp Infection.

## References

[1] Burk R., Bollinger L., Johnson J.C., Wada J., Radoshitzky S.R., Palacios G., Bavari S., Jahrling P.B., Kuhn J.H. (2016). Neglected filoviruses. FEMS Microbiol. Rev..

[2] Jacob S.T., Crozier I., Fischer W.A., Hewlett A., Kraft C.S., Vega M.A., Soka M.J., Wahl V., Griffiths A., Bollinger L. (2020). Ebola virus disease. Nat. Rev. Dis. Primers.

[3] Goeijenbier M., Van Kampen J., Reusken C., Koopmans M., Van Gorp E. (2014). Ebola virus disease: A review on epidemiology, symptoms, treatment and pathogenesis. Neth. J. Med..

[4] Breman J.G., Heymann D.L., Lloyd G., McCormick J.B., Miatudila M., Murphy F.A., Muyembé-Tamfun J.-J., Piot P., Ruppol J.-F., Sureau P. (2016). Discovery and description of Ebola Zaire virus in 1976 and relevance to the West African epidemic during 2013–2016. J. Infect. Dis..

[5] World Health Organization (WHO) Ebola—Democratic Republic of the Congo. https://www.who.int/emergencies/disease-outbreak-news/item/2021-DON325.

[6] World Health Organization (WHO) Ebola—Guinea. https://www.who.int/emergencies/disease-outbreak-news/item/2021-DON328.

[7] U.S. Food and Drug Administration First FDA-Approved Vaccine for the Prevention of Ebola Virus Disease, Marking a Critical Milestone in Public Health Preparedness and Response. https://www.fda.gov/news-events/press-announcements/first-fda-approved-vaccine-prevention-ebola-virus-disease-marking-critical-milestone-public-health.

[8] U.S. Food and Drug Administration FDA Approves First Treatment for Ebola Virus. https://www.fda.gov/news-events/press-announcements/fda-approves-first-treatment-ebola-virus.

[9] Lee J.E., Saphire E.O. (2009). Ebolavirusglycoprotein structure and mechanism of entry. Future Virol..

[10] Nanbo A., Imai M., Watanabe S., Noda T., Takahashi K., Neumann G., Halfmann P., Kawaoka Y. (2010). Ebolavirus is internalized into host cells via macropinocytosis in a viral glycoprotein-dependent manner. PLoS Pathog..

[11] Aleksandrowicz P., Marzi A., Biedenkopf N., Beimforde N., Becker S., Hoenen T., Feldmann H., Schnittler H.-J. (2011). Ebola virus enters host cells by macropinocytosis and clathrin-mediated endocytosis. J. Infect. Dis..

[12] Chandran K., Sullivan N.J., Felbor U., Whelan S.P., Cunningham J.M. (2005). Endosomal proteolysis of the Ebola virus glycoprotein is necessary for infection. Science.

[13] Côté M., Misasi J., Ren T., Bruchez A., Lee K., Filone C.M., Hensley L., Li Q., Ory D., Chandran K. (2011). Small molecule inhibitors reveal Niemann–Pick C1 is essential for Ebola virus infection. Nature.

[14] Carette J.E., Raaben M., Wong A.C., Herbert A.S., Obernosterer G., Mulherkar N., Kuehne A.I., Kranzusch P.J., Griffin A.M., Ruthel G. (2011). Ebola virus entry requires the cholesterol transporter Niemann–Pick C1. Nature.

[15] Miller E.H., Obernosterer G., Raaben M., Herbert A.S., Deffieu M.S., Krishnan A., Ndungo E., Sandesara R.G., Carette J.E., Kuehne A.I. (2012). Ebola virus entry requires the host-programmed recognition of an intracellular receptor. EMBO J..

[16] Woollard S.M., Kanmogne G.D. (2015). Maraviroc: A review of its use in HIV infection and beyond. Drug Des. Dev. Ther..

[17] Kang C., Syed Y.Y. (2020). Bulevirtide: First Approval. Drugs.

[18] Basu A., Li B., Mills D.M., Panchal R.G., Cardinale S.C., Butler M.M., Peet N.P., Majgier-Baranowska H., Williams J.D., Patel I. (2011). Identification of a small-molecule entry inhibitor for filoviruses. J. Virol..

[19] Basu A., Mills D.M., Mitchell D., Ndungo E., Williams J.D., Herbert A.S., Dye J.M., Moir D.T., Chandran K., Patterson J.L. (2015). Novel small molecule entry inhibitors of Ebola virus. J. Infect. Dis..

[20] Lin L.T., Hsu W.C., Lin C.C. (2014). Antiviral natural products and herbal medicines. J. Tradit. Complement. Med..

[21] Lin L.T., Noyce R.S., Pham T.N., Wilson J.A., Sisson G.R., Michalak T.I., Mossman K.L., Richardson C.D. (2010). Replication of subgenomic hepatitis C virus replicons in mouse fibroblasts is facilitated by deletion of interferon regulatory factor 3 and expression of liver-specific microRNA 122. J. Virol..

[22] Connor R.I., Chen B.K., Choe S., Landau N.R. (1995). Vpr is required for efficient replication of human immunodeficiency virus type-1 in mononuclear phagocytes. Virology.

[23] Hung T.C., Jassey A., Liu C.H., Lin C.J., Lin C.C., Wong S.H., Wang J.Y., Yen M.H., Lin L.T. (2019). Berberine inhibits hepatitis C virus entry by targeting the viral E2 glycoprotein. Phytomedicine.

[24] Reed L.J., Muench H. (1938). A Simple Method of Estimating Fifty Per Cent Endpoints. Am. J. Epidemiol..

[25] Taiwan Herbal Pharmacopeia 3rd Ed. Committee (2019). Taiwan Herbal Pharmacopeia.

[26] Hsu W.C., Chang S.P., Lin L.C., Li C.L., Richardson C.D., Lin C.C., Lin L.T. (2015). Limonium sinense and gallic acid suppress hepatitis C virus infection by blocking early viral entry. Antivir. Res..

[27] Hung T.C., Jassey A., Lin C.J., Liu C.H., Lin C.C., Yen M.H., Lin L.T. (2018). Methanolic Extract of Rhizoma Coptidis Inhibits the Early Viral Entry Steps of Hepatitis C Virus Infection. Viruses.

[28] Lin L.T., Chen T.Y., Chung C.Y., Noyce R.S., Grindley T.B., McCormick C., Lin T.C., Wang G.H., Lin C.C., Richardson C.D. (2011). Hydrolyzable tannins (chebulagic acid and punicalagin) target viral glycoprotein-glycosaminoglycan interactions to inhibit herpes simplex virus 1 entry and cell-to-cell spread. J. Virol..

[29] Wang J.Y., Lin C.J., Liu C.H., Lin L.T. (2019). Use of Viral Entry Assays and Molecular Docking Analysis for the Identification of Antiviral Candidates against Coxsackievirus A16. J. Vis. Exp..

[30] Tai C.J., Li C.L., Tai C.J., Wang C.K., Lin L.T. (2015). Early Viral Entry Assays for the Identification and Evaluation of Antiviral Compounds. J. Vis. Exp..

[31] Maruyama T., Rodriguez L.L., Jahrling P.B., Sanchez A., Khan A.S., Nichol S.T., Peters C., Parren P.W., Burton D.R. (1999). Ebola virus can be effectively neutralized by antibody produced in natural human infection. J. Virol..

[32] Shedlock D.J., Bailey M.A., Popernack P.M., Cunningham J.M., Burton D.R., Sullivan N.J. (2010). Antibody-mediated neutralization of Ebola virus can occur by two distinct mechanisms. Virology.

[33] Shrivastava-Ranjan P., Flint M., Bergeron É., McElroy A.K., Chatterjee P., Albariño C.G., Nichol S.T., Spiropoulou C.F. (2018). Statins suppress Ebola virus infectivity by interfering with glycoprotein processing. MBio.

[34] McMullan L.K., Flint M., Dyall J., Albarino C., Olinger G.G., Foster S., Sethna P., Hensley L.E., Nichol S.T., Lanier E.R. (2016). The lipid moiety of brincidofovir is required for in vitro antiviral activity against Ebola virus. Antivir. Res..

[35] Albarino C.G., Wiggleton Guerrero L., Lo M.K., Nichol S.T., Towner J.S. (2015). Development of a reverse genetics system to generate a recombinant Ebola virus Makona expressing a green fluorescent protein. Virology.

[36] Younan P., Santos R.I., Ramanathan P., Iampietro M., Nishida A., Dutta M., Ammosova T., Meyer M., Katze M.G., Popov V.L. (2019). Ebola virus-mediated T-lymphocyte depletion is the result of an abortive infection. PLoS Pathog..

[37] Fischer W.A., Vetter P., Bausch D.G., Burgess T., Davey R.T., Fowler R., Hayden F.G., Jahrling P.B., Kalil A.C., Mayers D.L. (2018). Ebola virus disease: An update on post-exposure prophylaxis. Lancet Infect. Dis..

[38] Qian K., Morris-Natschke S.L., Lee K.H. (2009). HIV entry inhibitors and their potential in HIV therapy. Med. Res. Rev..

[39] Esté J.A., Telenti A. (2007). HIV entry inhibitors. Lancet.

[40] Tilton J.C., Doms R.W. (2010). Entry inhibitors in the treatment of HIV-1 infection. Antivir. Res..

[41] Goujon C., Moncorgé O., Bauby H., Doyle T., Ward C.C., Schaller T., Hué S., Barclay W.S., Schulz R., Malim M.H. (2013). Human MX2 is an interferon-induced post-entry inhibitor of HIV-1 infection. Nature.

[42] Majmudar H., Hao M., Sankaranarayanan N.V., Zanotti B., Volin M.V., Desai U.R., Tiwari V. (2019). A synthetic glycosaminoglycan mimetic blocks HSV-1 infection in human iris stromal cells. Antivir. Res..

[43] Liu S., Li R., Zhang R., Chan C.C., Xi B., Zhu Z., Yang J., Poon V.K., Zhou J., Chen M. (2011). CL-385319 inhibits H5N1 avian influenza A virus infection by blocking viral entry. Eur. J. Pharmacol..

[44] Waldmann M., Jirmann R., Hoelscher K., Wienke M., Niemeyer F.C., Rehders D., Meyer B. (2014). A nanomolar multivalent ligand as entry inhibitor of the hemagglutinin of avian influenza. J. Am. Chem. Soc..

[45] Schmidt A.G., Lee K., Yang P.L., Harrison S.C. (2012). Small-molecule inhibitors of dengue-virus entry. PLoS Pathog..

[46] Kuo Y.T., Liu C.H., Li J.W., Lin C.J., Jassey A., Wu H.N., Perng G.C., Yen M.H., Lin L.T. (2020). Identification of the phytobioactive Polygonum cuspidatum as an antiviral source for restricting dengue virus entry. Sci. Rep..

[47] Syder A.J., Lee H., Zeisel M.B., Grove J., Soulier E., Macdonald J., Chow S., Chang J., Baumert T.F., McKeating J.A. (2011). Small molecule scavenger receptor BI antagonists are potent HCV entry inhibitors. J. Hepatol..

[48] Lin L.T., Chung C.Y., Hsu W.C., Chang S.P., Hung T.C., Shields J., Russell R.S., Lin C.C., Li C.F., Yen M.H. (2015). Saikosaponin b2 is a naturally occurring terpenoid that efficiently inhibits hepatitis C virus entry. J. Hepatol..

[49] Chung C.Y., Liu C.H., Burnouf T., Wang G.H., Chang S.P., Jassey A., Tai C.J., Tai C.J., Huang C.J., Richardson C.D. (2016). Activity-based and fraction-guided analysis of Phyllanthus urinaria identifies loliolide as a potent inhibitor of hepatitis C virus entry. Antivir. Res..

[50] Lin L.T., Chen T.Y., Lin S.C., Chung C.Y., Lin T.C., Wang G.H., Anderson R., Lin C.C., Richardson C.D. (2013). Broad-spectrum antiviral activity of chebulagic acid and punicalagin against viruses that use glycosaminoglycans for entry. BMC Microbiol..

[51] Ha M.N., Delpeut S., Noyce R.S., Sisson G., Black K.M., Lin L.T., Bilimoria D., Plemper R.K., Prive G.G., Richardson C.D. (2017). Mutations in the Fusion Protein of Measles Virus that Confer Resistance to the Membrane Fusion Inhibitors Carbobenzoxy-d-Phe-l-Phe-Gly and 4-Nitro-2-Phenylacetyl Amino-Benzamide. J. Virol..

[52] Lin C.-J., Liu C.-H., Wang J.Y., Lin C.-C., Li Y.-F., Richardson C.D., Lin L.-T. (2018). Small molecules targeting coxsackievirus A16 capsid inactivate viral particles and prevent viral binding. Emerg. Microbes Infect..

[53] De Colibus L., Wang X., Tijsma A., Neyts J., Spyrou J.A., Ren J., Grimes J.M., Puerstinger G., Leyssen P., Fry E.E. (2015). Structure Elucidation of Coxsackievirus A16 in Complex with GPP3 Informs a Systematic Review of Highly Potent Capsid Binders to Enteroviruses. PLoS Pathog..

[54] Haqqani A.A., Tilton J.C. (2013). Entry inhibitors and their use in the treatment of HIV-1 infection. Antivir. Res..

[55] Xiao F., Fofana I., Thumann C., Mailly L., Alles R., Robinet E., Meyer N., Schaeffer M., Habersetzer F., Doffoël M. (2015). Synergy of entry inhibitors with direct-acting antivirals uncovers novel combinations for prevention and treatment of hepatitis C. Gut.

[56] Corti D., Misasi J., Mulangu S., Stanley D.A., Kanekiyo M., Wollen S., Ploquin A., Doria-Rose N.A., Staupe R.P., Bailey M. (2016). Protective monotherapy against lethal Ebola virus infection by a potently neutralizing antibody. Science.

[57] Qiu X., Wong G., Audet J., Bello A., Fernando L., Alimonti J.B., Fausther-Bovendo H., Wei H., Aviles J., Hiatt E. (2014). Reversion of advanced Ebola virus disease in nonhuman primates with ZMapp. Nature.

[58] Mulangu S., Dodd L.E., Davey R.T., Tshiani Mbaya O., Proschan M., Mukadi D., Lusakibanza Manzo M., Nzolo D., Tshomba Oloma A., Ibanda A. (2019). A Randomized, Controlled Trial of Ebola Virus Disease Therapeutics. N. Engl. J. Med..

[59] Cui Q., Cheng H., Xiong R., Zhang G., Du R., Anantpadma M., Davey R.A., Rong L. (2018). Identification of diaryl-quinoline compounds as entry inhibitors of Ebola virus. Viruses.

[60] Shoemaker C.J., Schornberg K.L., Delos S.E., Scully C., Pajouhesh H., Olinger G.G., Johansen L.M., White J.M. (2013). Multiple cationic amphiphiles induce a Niemann-Pick C phenotype and inhibit Ebola virus entry and infection. PLoS ONE.

[61] Cheng H., Lear-Rooney C.M., Johansen L., Varhegyi E., Chen Z.W., Olinger G.G., Rong L. (2015). Inhibition of Ebola and Marburg Virus Entry by G Protein-Coupled Receptor Antagonists. J. Virol..

[62] Qiu X., Kroeker A., He S., Kozak R., Audet J., Mbikay M., Chretien M. (2016). Prophylactic Efficacy of Quercetin 3-beta-O-d-Glucoside against Ebola Virus Infection. Antimicrob. Agents Chemother..

[63] Chen Q., Tang K., Guo Y. (2020). Discovery of sclareol and sclareolide as filovirus entry inhibitors. J. Asian Nat. Prod. Res..

[64] Alvarez C.P., Lasala F., Carrillo J., Muniz O., Corbi A.L., Delgado R. (2002). C-type lectins DC-SIGN and L-SIGN mediate cellular entry by Ebola virus in cis and in trans. J. Virol..

[65] Kondratowicz A.S., Lennemann N.J., Sinn P.L., Davey R.A., Hunt C.L., Moller-Tank S., Meyerholz D.K., Rennert P., Mullins R.F., Brindley M. (2011). T-cell immunoglobulin and mucin domain 1 (TIM-1) is a receptor for Zaire Ebolavirus and Lake Victoria Marburgvirus. Proc. Natl. Acad. Sci. USA.

[66] Simmons G., Reeves J.D., Grogan C.C., Vandenberghe L.H., Baribaud F., Whitbeck J.C., Burke E., Buchmeier M.J., Soilleux E.J., Riley J.L. (2003). DC-SIGN and DC-SIGNR bind ebola glycoproteins and enhance infection of macrophages and endothelial cells. Virology.

[67] Shimojima M., Ikeda Y., Kawaoka Y. (2007). The mechanism of Axl-mediated Ebola virus infection. J. Infect. Dis..

[68] Brindley M.A., Hunt C.L., Kondratowicz A.S., Bowman J., Sinn P.L., McCray P.B., Quinn K., Weller M.L., Chiorini J.A., Maury W. (2011). Tyrosine kinase receptor Axl enhances entry of Zaire ebolavirus without direct interactions with the viral glycoprotein. Virology.

[69] Takada A., Watanabe S., Ito H., Okazaki K., Kida H., Kawaoka Y. (2000). Downregulation of beta1 integrins by Ebola virus glycoprotein: Implication for virus entry. Virology.

[70] Chan S.Y., Empig C.J., Welte F.J., Speck R.F., Schmaljohn A., Kreisberg J.F., Goldsmith M.A. (2001). Folate receptor-alpha is a cofactor for cellular entry by Marburg and Ebola viruses. Cell.

[71] Shimojima M., Takada A., Ebihara H., Neumann G., Fujioka K., Irimura T., Jones S., Feldmann H., Kawaoka Y. (2006). Tyro3 family-mediated cell entry of Ebola and Marburg viruses. J. Virol..

[72] Brudner M., Karpel M., Lear C., Chen L., Yantosca L.M., Scully C., Sarraju A., Sokolovska A., Zariffard M.R., Eisen D.P. (2013). Lectin-dependent enhancement of Ebola virus infection via soluble and transmembrane C-type lectin receptors. PLoS ONE.

[73] Saeed M.F., Kolokoltsov A.A., Albrecht T., Davey R.A. (2010). Cellular entry of ebola virus involves uptake by a macropinocytosis-like mechanism and subsequent trafficking through early and late endosomes. PLoS Pathog..

[74] Samuel C.E. (2001). Antiviral Actions of Interferons. Clin. Microbiol. Rev..

[75] Prins K.C., Delpeut S., Leung D.W., Reynard O., Volchkova V.A., Reid S.P., Ramanan P., Cardenas W.B., Amarasinghe G.K., Volchkov V.E. (2010). Mutations abrogating VP35 interaction with double-stranded RNA render Ebola virus avirulent in guinea pigs. J. Virol..

[76] Lee J.H., Park K.H., Lee M.-H., Kim H.-T., Seo W.D., Kim J.Y., Baek I.-Y., Jang D.S., Ha T.J. (2013). Identification, characterisation, and quantification of phenolic compounds in the antioxidant activity-containing fraction from the seeds of Korean perilla (*Perilla frutescens*) cultivars. Food Chem..

[77] Zhou X.-J., Yan L.-L., Yin P.-P., Shi L.-L., Zhang J.-H., Liu Y.-J., Ma C. (2014). Structural characterisation and antioxidant activity evaluation of phenolic compounds from cold-pressed Perilla frutescens var. arguta seed flour. Food Chem..

