# Supplementary material for: The Methanolic Extract of Perilla frutescens Robustly Restricts Ebola Virus Glycoprotein-Mediated Entry
Source: Viruses. 2021 Sep 8;13(9):1793. doi: 10.3390/v13091793 (PMC8473196; doi:10.3390/v13091793)
Supplement: Supplementary file 1 [file viruses-13-01793-s001.zip › viruses-1342630-si final.pdf]

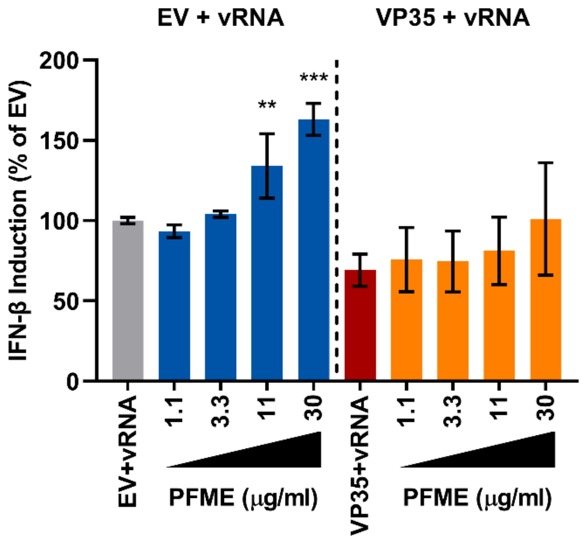

Supplementary Figure S1. PFME Induces IFN-β Promoter Activation That Can Be Suppressed by the Overexpression of the IFN-β Antagonist EBOV VP35. The pcDNA3-EBOV VP35, carrying EBOV VP35 gene form Zaire species (1976 Yambuku-Mayinga strain) was previously described [1]. Luciferase Reporter Gene Assay was adapted from [2]. Briefly, HEK 293T cells (1.5×10^4 cells/well of 96-well plates) were seeded overnight and transfected (T-Pro P-Fect Transfection Reagent; T-Pro Biotechnology, New Taipei City, Taiwan) with the respective plasmids (IFN-β promoter reporter pGL-IFN-β-luc 60 ng; Renilla control pRL-TK 10 ng; 0.012 ng of pcDNA3 VP35 encoding the EBOV VP35 or pcDNA3 that was used as empty vector [EV] in controls). Twenty-four hours after transfection, cells were stimulated with 200 ng/well of influenza A virus (IAV)-RNA (prepared as previously reported [1]), pre-mixed with the transfection reagent in reduced serum medium Opti-Mem (GIBCO/ThermoFisher Scientific), in the presence of different concentrations of PFME and further incubated for 24 h before harvesting the cells and assessing for luciferase activity. The relative light units (RLU) of luciferase reporter activity were normalized to Renilla Luc and over unstimulated controls. Each assay was performed in triplicate. Results were expressed in percentage of IFN induction over the empty vector (indicated as 100%) and plotted in mean ± SD. Data were analyzed with one-way ANOVA. Asterisks (*) denote statistical significance: $**p < 0.01$; $***p < 0.001$.

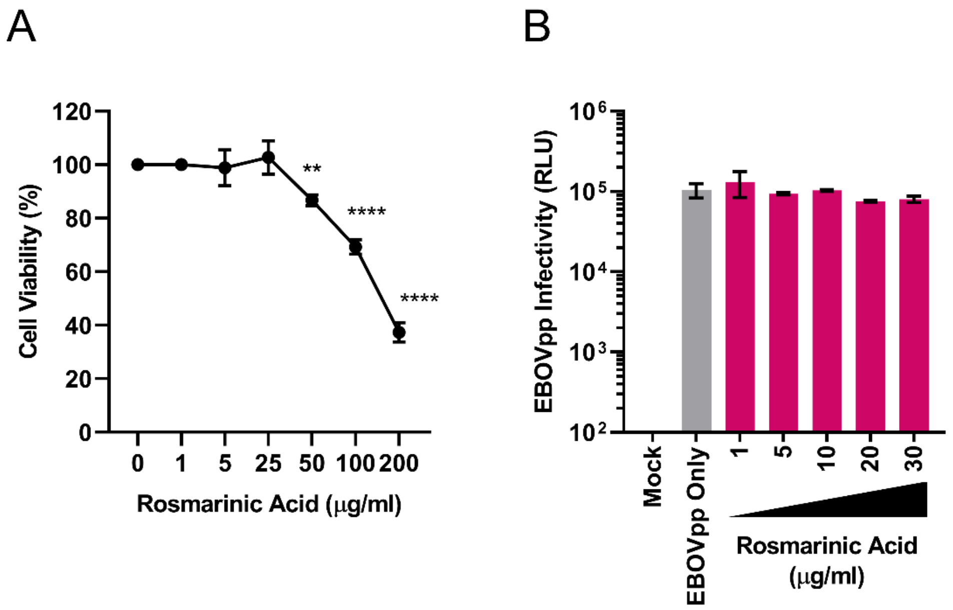

Supplementary Figure S2. Rosmarinic Acid Does Not Impede EBOVpp Infection. (A) Cytotoxicity of rosmarinic acid on Huh-7 cells. (B) Huh-7 cells (1×10^5 cells/well seeded in 48-well plates) were pre-incubated with rosmarinic acid (Sigma) at the indicated doses for 24 h, and then infected with EBOVpp (MOI 0.01) in the presence of the compound again at the same concentration range. After 2 h infection at 37 °C, the cells were washed with DPBS and further incubated in basal media for 72 h at 37 °C, before luciferase assay was performed as described in the text. Data are expressed as mean RLU ± SD from 3 independent repeats. Asterisks (*) denote statistical significance: **$p < 0.01$; ****$p < 0.0001$.

References

1. Cannas, V.; Daino, G.L.; Corona, A.; Esposito, F.; Tramontano, E. A Luciferase Reporter Gene Assay to Measure Ebola Virus Viral Protein 35-Associated Inhibition of Double-Stranded RNA-Stimulated, Retinoic Acid-Inducible Gene 1-Mediated Induction of Interferon beta. *J Infect Dis* **2015**, *212 Suppl 2*, S277-281, doi:10.1093/infdis/jiv214.
2. Fanunza, E.; Frau, A.; Sgarbanti, M.; Orsatti, R.; Corona, A.; Tramontano, E. Development and Validation of a Novel Dual Luciferase Reporter Gene Assay to Quantify Ebola Virus VP24 Inhibition of IFN Signaling. *Viruses* **2018**, *10*, doi:10.3390/v10020098.
